# Can selenium deficiency in Malawi be alleviated through consumption of agro-biofortified maize flour? Study protocol for a randomised, double-blind, controlled trial

**DOI:** 10.1186/s13063-019-3894-2

**Published:** 2019-12-30

**Authors:** Edward J. M. Joy, Alexander A. Kalimbira, Dawd Gashu, Elaine L. Ferguson, Joanna Sturgess, Alan D. Dangour, Leonard Banda, Gabriella Chiutsi-Phiri, Elizabeth H. Bailey, Simon C. Langley-Evans, R. Murray Lark, Kate Millar, Scott D. Young, Limbanazo Matandika, Joseph Mfutso-Bengo, John C. Phuka, Felix P. Phiri, Jellita Gondwe, E. Louise Ander, Nicola M. Lowe, Patson C. Nalivata, Martin R. Broadley, Elizabeth Allen

**Affiliations:** 1 Faculty of Epidemiology and Population Health, London School of Hygiene & Tropical Medicine, Keppel Street, London, WC1E 7HT UK; 2 London Centre for Integrative Research on Agriculture and Health, London School of Hygiene & Tropical Medicine, Keppel Street, London, WC1E 7HT UK; 3 Lilongwe University of Agriculture and Natural Resources, PO BOX 219, Bunda College, Lilongwe, Malawi; 4 Center for Food Science and Nutrition, Addis Ababa University, Addis Ababa, Ethiopia; 5 Lilongwe University of Agriculture and Natural Resources, PO BOX 143, Natural Resources College, Lilongwe, Malawi; 6 School of Biosciences, University of Nottingham, Sutton Bonington Campus, Loughborough, LE12 5RD UK; 7 School of Public Health and Family Medicine, College of Medicine, University of Malawi, Blantyre, Malawi; 8 Department of Nutrition, HIV and AIDS, Ministry of Health, Lilongwe, Malawi; 9 Public Health Institute of Malawi, Community Health Sciences Unit, National Nutrition Reference Lab, Lilongwe, Malawi; 10 Inorganic Geochemistry, Centre for Environmental Geochemistry, British Geological Survey, Nottingham, NG12 5GG UK; 11 UCLan Research Centre for Global Development, University of Central Lancashire, Preston, PR1 2HE UK

**Keywords:** Biofortification, Fertilisers, Malawi, Micronutrients, Nutrition, Selenium, Trial

## Abstract

**Background:**

Micronutrient deficiencies including selenium (Se) are widespread in Malawi and potentially underlie a substantial disease burden, particularly among poorer and marginalised populations. Concentrations of Se in staple cereal crops can be increased through application of Se fertilisers – a process known as agronomic biofortification (agro-biofortification) – and this may contribute to alleviating deficiencies. The Addressing Hidden Hunger with Agronomy (AHHA) trial aims to establish the efficacy of this approach for improving Se status in rural Malawi.

**Methods:**

A double-blind, randomised, controlled trial will be conducted in a rural community in Kasungu District, Central Region, Malawi. The hypothesis is that consumption of maize flour agro-biofortified with Se will increase serum Se concentration. We will recruit 180 women of reproductive age (WRA) (20–45 years) and 180 school-age children (SAC) (5–10 years) randomly assigned in a 1:1 ratio to receive either maize flour enriched through agro-biofortification with Se or a control flour not enriched with Se. Households will receive flour (330 g per capita per day) for 12 weeks. The primary outcome is Se concentration in serum (μg/L). Serum will be extracted from venous blood samples drawn at baseline (prior to flour distribution) and end-line. Selenium concentration will be measured by using inductively coupled plasma mass spectrometry.

**Discussion:**

Findings will be communicated to policy stakeholders and participating communities and reported in peer-reviewed journals.

**Trial registration:**

The Addressing Hidden Hunger with Agronomy (Malawi) trial is registered (5^th^ March 2019; ISCRTN85899451).

## Background

Micronutrient deficiencies are widespread in Malawi [1]. Selenium (Se) is an essential micronutrient with important roles in thyroid function and cognitive development and as a component of selenoprotein anti-oxidants [2, 3]. In a recent study of a nationally representative sample of Malawian women and pre-school children, plasma Se concentrations were below the threshold for optimal activity of the anti-oxidant enzyme glutathione peroxidase 3 (GPx3) in 62% of women (15–49 years; *n* = 754) and 86% of pre-school children (6–59 months; *n* = 990) [4]. Deficiency of Se occurs because of inadequate dietary Se intakes and this is driven by low concentrations of plant-available Se in the weathered agricultural soils that are typical of Malawi [5–7].

There are various potential strategies – including dietary diversification, food fortification at processing stage, and supplementation – to alleviate Se deficiencies. Alternatively, the concentration of bioavailable Se can be increased in the edible portion of staple crops – a process known as biofortification. For some micronutrients, biofortification can be achieved through crop breeding [8], but agro-biofortification (i.e., application of micronutrient fertilisers) is required for Se because of the dominance of environmental over genetic controls of Se concentration in crops [9]. Agro-biofortification of maize can increase concentrations of Se in grain and may be a cost-effective and equitable strategy in Malawi, where maize contributes 60% of dietary energy supply and more among poorer populations [5, 7].

Agro-biofortification with Se has policy precedent in Finland, where incorporation of Se into granular fertilisers has been mandatory since 1984. Mean plasma Se concentrations of adults (*n* = 60) increased from 70.3 μg/L prior to the fertiliser policy change in 2010 to 110.5 μg/L, which is considered optimal status [10]. However, this strategy has not been implemented in low- to middle-income countries where agriculture, food systems and dietary patterns are substantially different from those in high-income countries.

This study aims to test the efficacy of improving Se status among a rural Malawian population through consumption of maize flour enriched with Se through agro-biofortification. The evidence may inform fertiliser policies in Malawi.

## Methods and design

The study protocol is reported according to SPIRIT guidelines [11] (Additional file 1).

### Study setting

The Addressing Hidden Hunger with Agronomy (AHHA) Malawi trial will be conducted in Wimbe Traditional Authority (TA), Kasungu District, Central Region, Malawi. The study area was selected because of the high prevalence of Se deficiency [4]. Most households rely on subsistence farming alongside smallholder and estate tobacco production [12]. To define a study area, two neighbouring enumeration areas (EAs) were randomly selected from a total of 51 EAs in Wimbe TA; EAs are the primary sampling unit of the national Demographic and Health Survey (DHS). We conducted a census to create a list of all households in the EA (*n* = 1179) and a roster of all household members.

### Study design

**Fig. 1:**
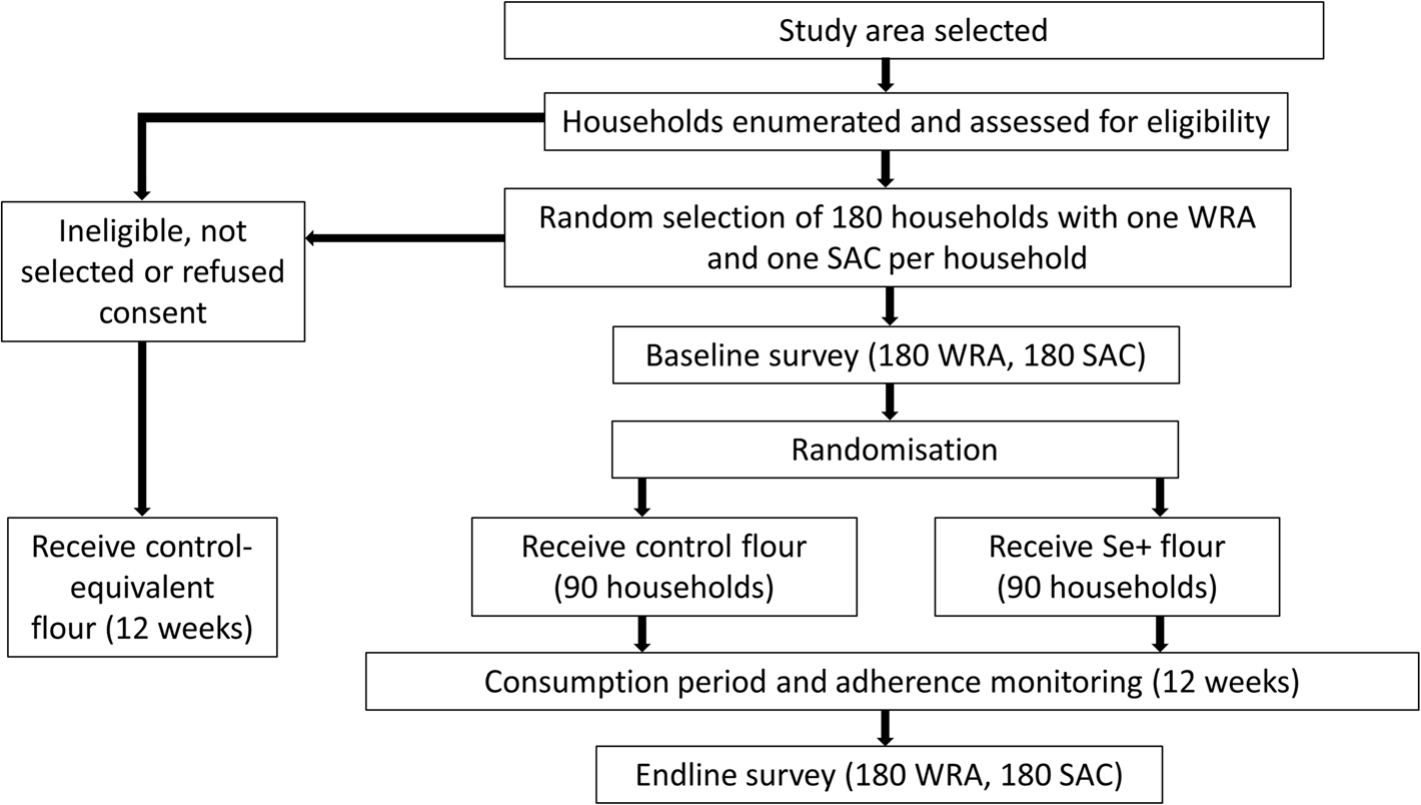
CONSORT diagram of the Addressing Hidden Hunger with Agronomy (AHHA) Malawi trial. The trial aims to recruit 180 school-age children (SAC) and 180 women of reproductive age (WRA)

The AHHA Malawi trial is a two-arm randomised, double-blind, controlled trial with participants receiving maize flour enriched through agro-biofortification with Se or a control flour that is not enriched with Se (Fig. 1).

Prior to baseline assessments, 180 households will be randomly selected from eligible households (see ‘Inclusion criteria’). An additional 50 households will be pre-selected in case of consent refusals. Households will be visited for recruitment of one non-pregnant woman of reproductive age (WRA) (20–45 years) and one school-age child (SAC) (5–10 years). Recruitment and baseline questionnaires will be conducted in the household and participants will then be directed to nearby mobile field clinics set up in large tents where anthropometry and blood sampling will be conducted. Recruitment will be conducted by trained research assistants (RAs) hired by Lilongwe University of Agriculture and Natural Resources (LUANAR). The trial aims to recruit 180 WRA and 180 SAC.

After baseline, households will be randomly allocated in a 1:1 ratio to receive Se flour (*n* = 90) or control flour (*n* = 90). The trial statistician will allocate households remotely by using a computer-based randomisation programme. Intervention allocation will be concealed from participants, RAs conducting the baseline and end-line surveys, laboratory analysts and those involved in managing or overseeing the trial. Locally, only the supervisor of the maize flour distribution will know the allocation of households to treatments.

### Inclusion criteria

Household level:

- Household members include at least one non-pregnant WRA (age 20–45 years) and at least one SAC (age 5–10 years) in residence during July to October.
- Household typically prepares and consumes meals at home.
- Household head agrees that the household will receive and all members consume maize flour in place of their own flour for the 12-week flour distribution period.

Individual level:

- One non-pregnant WRA (age 20–45 years) and one SAC (age 5–10 years) per household. Pregnancy status is self-reported.
- Participant is planning to be in residence in the household during July to October.
- Participant WRA is willing and able to provide consent, and the caretaker of the participant SAC is willing and able to provide assent.

### Formative work

The trial delivery team have undertaken substantial preparatory work with the community. Formative work was conducted from July to September 2018 to identify potential concerns among community members about participation in the trial and to develop mitigating strategies. Community engagement activities have been conducted and these include briefings with traditional leaders and relevant government officials, question-and-answer sessions with each village in the study area, and a community visit to the maize production site.

### Description of treatment

**Fig. 2:**
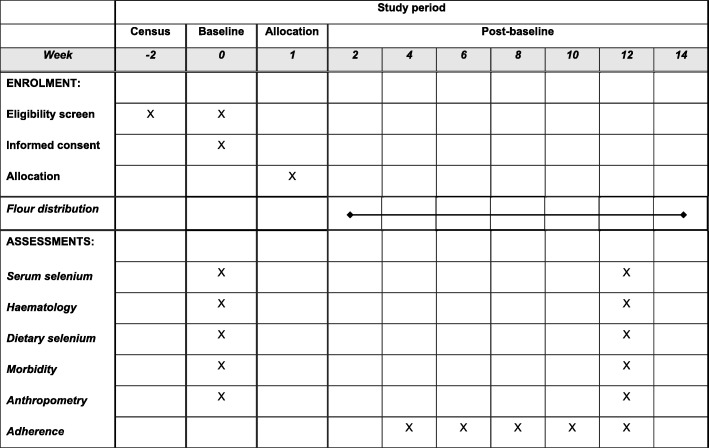
Schedule of enrolment, interventions, and assessments for the Addressing Hidden Hunger with Agronomy Malawi trial

The flour distribution period will last 12 weeks during July to October 2019. The intervention will begin 1–2 weeks after the baseline assessment is complete. End-line blood samples will be taken 1–2 weeks prior to the final flour distribution (Fig. 2).

Maize flour will be distributed to intervention and control households in multiples of 5- and 10 kg sacks at pre-determined distribution points set up in the vicinity of the villages. Households will receive sufficient maize flour to meet requirements for the 12-week flour distribution period with distributions at six time points. Household requirements will be calculated on the basis of estimated consumption of 330 g/capita/day for all household members over the age of 1 year (equivalent to 10 kg /capita/month).

The provision of non-biofortified flour to control households allows the trial to be blinded. However, bags of maize flour will be labelled to ensure that households receive the correct treatment and to monitor consumption. The trial statistician will generate a set of unique seven-digit codes that will be used to label the bags of study flour. The statistician will communicate the treatment codes directly to the maize flour distribution team lead. Only the trial statistician and maize distribution team will know how to interpret the code. The rest of the trial team and participants will be blinded to treatment allocation. At distribution points, study flour bags will be stacked in piles according to the bag code. Participants will present their ID cards (Additional file 2i) and the maize distribution team will check their allocated treatment before providing the correct quantity and type of flour. Participants will receive help transporting maize flour to their home if necessary.

Non-participating households in the study area will also receive non-biofortified flour for the duration of the study. Control-equivalent flour will not be labelled with codes and will be distributed separately. The provision of free flour to non-participant households was deemed appropriate to prevent individuals from feeling coerced into participating in the trial, to avoid envy or stigma within the community, and to reduce the likelihood of sharing flour between households.

The trial maize was grown at LUANAR, Bunda Campus, Malawi. A widely grown hybrid cultivar (DKC80–53) was grown by using conventional agronomy. Plants were spaced at 0.25 m within rows; the distance between rows was 0.75 m. When the crop was approximately 1–1.5 m in height, at the pre-tasselling stage, sodium selenate solution was sprayed continuously along the tops of ridges (stem bases) at 18 g Se/ha (elemental basis) applied by using knapsack sprayers and an application volume of 100 L water/ha [13]. Maize grain was sampled when the crop was mature, about 1 month prior to harvest, and analysed by inductively coupled plasma mass spectrometry (ICPMS). At harvest, the mean concentrations of Se were 0.010 mg/kg in control grain and 0.279 mg/kg in Se-biofortified grain. The Se-biofortified grain will be blended in a 2:1 ratio with control grain to obtain a concentration of 0.2 mg/kg, the target for the intervention flour. In comparison, the median Se concentration in maize grain samples from farmer fields on non-calcareous soil types in Malawi was 0.015 mg/kg (*n* = 105) [5, 7, 14]. Grain will be milled and packed in labelled sacks. Milling equipment will be thoroughly cleaned between processing the Se-biofortified and control grain.

Maize flour in Malawi comes in three main types: *ufa mgaiwa* (unrefined), *ufa granmill* (partly refined with bran removed) or *ufa woyera* (fully refined with bran and endosperm removed). We will provide *ufa granmill* during the trial as this was the preferred type of flour among participants in formative research.

### Objectives

The objective of the study is to test whether the Se status of non-pregnant WRA and SAC can be improved through consumption of maize flour enriched with Se through agro-biofortification. We hypothesise that dietary Se intake will be substantially greater among those receiving Se-biofortified maize flour compared with control flour and that this will translate into increased Se status among those receiving fortified maize measured as serum Se concentration.

Initially, the trial was designed to test the efficacy of alleviating Se and zinc (Zn) deficiency (May 2019; ISRCTN85899451). However, in production of the maize for the study, the target maize Zn concentration was not reached and this was likely due to a combination of soil factors and heavy rainfall during the 2018/19 production season. Therefore, the planned Zn arm of the trial was underpowered and dropped for ethical reasons.

### Outcome measures

#### Primary outcome measure

##### Serum Se concentration

Serum Se concentrations will be measured at baseline and end-line. Whole venous blood will be drawn into trace element–free tubes (BD Diagnostics, Eysins, Switzerland); after clotting, the serum will be separated by centrifugation within 40 min. Serum 0.5-mL aliquots will be transferred into cryovials, transported in mobile freezers and stored at −80 °C. Serum samples will be shipped on dry ice to the University of Nottingham, UK, for determination of elemental Se concentration by ICPMS [4]. The unit of measure is concentration of Se in serum (μg/L).

#### Secondary outcomes

The prevalence of Se deficiency will be compared between treatment groups. Deficiency will be defined by using established thresholds for the optimal activity of GPx3 (<84.9 μg/L) and iodothyronine deiodinase (IDI) (<64.8 μg/L) and for Keshan disease (<30 μg/L), which is a cardiomyopathy linked to Se status reported in China [15].

Concentration in serum of the markers of inflammation alpha (1)-acid glycoprotein (AGP) (mg/L) and C-reactive protein (CRP) (mg/L) will be measured by using a sandwich enzyme-linked immunosorbent assay (ELISA). Concentration of haemoglobin in whole venous blood (g/dL) will be measured in the field by trained nurses using a portable HemoCue Hb201^+^ (HemoCue AB, Ängelholm, Sweden).

The prevalence of anaemia will be compared between treatment groups. Anaemia will be defined by using standard thresholds [16].

Dietary Se intakes will be quantified among WRA. Dietary data will be collected among participating WRA using four-pass interactive 24-h recall. Two days before the 24-h recall, participants will be given plates/bowls and asked to consume foods on a plate separate from other family members. In the first pass, participants will be asked to recall all foods and beverages they consumed over the previous day (from midnight to midnight). In the second pass, they will be asked details about the foods/beverages and time/place of consumption. In the third pass, they will be asked to estimate the quantity of each food/beverage consumed by using an interviewer-assisted method (e.g., food models and diet scales) and the source of each food (i.e., home produced, purchased, the AHHA project, from neighbours/relatives, gifted, food aid/food for work or wild). In the fourth pass, the recalled intake over the day will be reviewed. Dietary data will be collected across all days of the week (population level) to account for any day-of-the-week effect. A repeat 24-h recall will be collected from a sub-sample of participants (*n* = 60) at least 2 days after the initial recall. Dietary data will be combined with relevant food composition data [7] to determine the baseline intakes of energy and micronutrients. The percentage of women at risk of inadequate intakes of Se will be estimated by using the Estimated Average Requirement (EAR) cut-point method [17] after adjustment for intra-subject variability. The percentage of energy from food groups, sub-groups and specific foods will be estimated to examine changes in diet patterns.

Morbidity outcomes will be self-reported at baseline and end-line by using standard questions from the Malawi DHS. Diarrhoea incidence, severity and duration, incidence of vomiting and incidence of fever will be recorded for WRA and SAC. Prevalence of pneumonia will be self-reported among SAC only (as per the DHS) (i.e., number of days of coughing and fast or difficult breathing (due to a problem in the chest) in the two weeks prior to the survey).

#### Monitoring of adherence

Participant consumption of flour will be monitored during the course of the intervention. Households will be visited once every 15 days. RAs will observe the number of 10 kg bags of study flour that have not been consumed and will count and collect the number of empty bags of study flour for reconciliation. A household member responsible for meal preparation will be asked what flour was used the previous day for each meal and what quantity of non-study flour was used by the household over the past two weeks. The same member of the household will be asked how many bags of study flour have been sold or gifted away. Combined, these data will provide a proxy measure of household consumption. Data collected for the monitoring of adherence will not be used to alter the amount of study flour received by participating households at future distribution time points.

#### Monitoring of participant safety

Consumption of 330 g/capita/day of the agro-biofortified maize flour with Se concentration of about 0.3 mg/kg would supply 99 μg Se/capita/day. The adult recommended dietary allowance for Se is 55 μg/capita/day and the tolerable upper level of intake is 400 μg/day [18]. The concentration of Se in maize grain depends largely on soil factors, and the concentration in Se-biofortified grain (mean 0.279 mg/kg) produced for this study is within the “natural” range of maize grain Se concentrations observed previously in Malawi (0.005–0.533 mg/kg) [5]. Comparatively greater Se concentrations occur naturally (i.e., without addition of Se fertiliser) in maize grown on Eutric Vertisol soil types, found in southern Malawi in the Shire Valley and along the shore of Lake Malawi.

Consumption of Se-biofortified flour in the quantities provided in this study is not known or expected to cause adverse events (AEs). Procedures will be set up to capture and monitor safety. Non-serious AEs will be captured and recorded in electronic monitoring forms by RAs during adherence monitoring visits to participating households (every 15 days). RAs will ask participants and caregivers of children about any AEs, including fever, diarrhoea and coughing. These will be compiled and reported to the principal investigator (PI) on a monthly basis.

Serious adverse events (SAEs) include death, life-threatening illness, hospitalisation, persistent or significant disability, or other such occurrences, and these will be reported to the study coordination centre (LUANAR) through completion of an SAE form and submission within 24 h of the monitoring team being made aware of the event. Participants will be informed how to contact the study coordination centre; and RAs, health surveillance assistants and health volunteers will be trained on how to record and report SAEs. A medical doctor will be “on call” and available to visit participants through the duration of the intervention to advise on seriousness and causality. The study PI (Edward Joy) will record the event and its seriousness, causality and expectedness.

The PI will report SAEs to the trial statistician who is aware of random treatment allocation and who will report to the independent statistician on the trial steering committee (TSC) to decide about the continuation of the trial. All AEs and SAEs will be compiled by the PI and reported to the research ethics committees (RECs) in accordance with their criteria.

Unblinding will occur only at the request of the independent statistician on the TSC. Unblinding would involve revealing the allocation of households to treatment (e.g., to investigate the occurrence of SAEs and their association with treatment). At this point, the TSC may request that the trial be paused or terminated.

### Withdrawal

Any individual will remain free to withdraw at any time from the study. If a participant withdraws consent from further trial participation, their data will remain on file and will be included in the final study analysis. If a participant withdraws consent for their data to be used, the data will be destroyed immediately.

### Loss to follow-up

Households or individuals that move out of the village will leave the trial and no attempt will be made to follow them up. Any data collected from participants who are lost to follow-up will be included in the analysis where possible. Continued community engagement will be conducted to minimise loss to follow-up. Contact details, including mobile phone numbers, have been recorded during household enumeration, and participants will be contacted 1–2 days before the mobile clinic is set up in their vicinity. Mobile clinics will be located close to participant households, and transport will be provided if participants require. Also, participants will be contacted 1–2 days prior to flour distribution.

### Data collection and management

All participants will be assigned a unique numeric ID that will be used in data capture forms and subsequent analyses to maintain anonymity. Questionnaire, anthropometry and adherence data will be collected by trained RAs in the field via passcode-protected tablets using an Open Data Kit (ODK) [19]. Blood samples will be taken by trained and experienced nurses and processed to serum by trained technicians. Sample tubes and subsequent data will be labelled with unique, anonymous codes. Samples will be logged in the field using ODK forms. Completed forms will be uploaded to a secure cloud server daily and will be encrypted for security. Mobile clinic team leads will report daily to the anthropometry and blood collection team supervisors to ensure that standard procedures are followed. The trial manager and data manager will reconcile samples and data forms daily. Recruitment rates and number of eligible individuals will be assessed and compared with numbers enrolled and completeness of follow-up.

Dietary data will be collected by trained RAs by using a paper-based data collection process. Dietary forms will be labelled with participant IDs. Data collection will be supervised by the diet team supervisor, who will observe individual 24-h recalls and check the data collected each day. The RA, supervisor and coordinator will sign off all forms before they are filed for data entry. Quality control checklists (for observations and forms) will be used to ensure that standard procedures are followed. At the end of the baseline and end-line surveys, all diet data will be double-entered to reduce errors and ensure that consistent data entry decisions are made.

The data manager will hold the data tables that match participant ID to sample codes, and these will not be available to the trial statistician until after statistical analyses are complete. Thus, the trial statistician will be blinded to treatments. Sample analysis will be undertaken by Malawian and UK study collaborators in laboratories in the UK. Good practices in quality control will be followed, such as analysis in duplicate and inclusion of sample blanks and reference materials and use of sufficiently sensitive analytical instrumentation to ensure quantification of the variables of interest.

All data collection and storage will be compliant with the General Data Protection Regulation [20] and the conditions of the REC approval. Anonymised data will be uploaded to an open data platform following analysis in compliance with open access data requirements. Data will be kept for a minimum of 10 years after completion of the trial.

### Biological sample management

Serum samples will be transported in cool boxes packed with ice from mobile field clinics to the Community Health Sciences Unit, Ministry of Health, Area 3, Lilongwe, where they will be stored at −80 °C. Cryovials and cryovial racks will be labelled with unique numeric and quick response (QR) codes. Instrumentation and methodologies are not established in Malawi for ICPMS to measure elemental concentrations in the parts-per-billion range. Samples will be transferred on dry ice to a secure laboratory at the University of Nottingham under a material transfer agreement (MTA) with appropriate licences. Samples will be cross-checked with shipment lists. Following ICPMS analysis, samples will be stored for a period of 5 years following completion of the trial after which they will be destroyed in accordance with licence and safety requirements.

### Statistical analysis

The trial is designed to have statistical power to detect an increase in Se concentration in serum of 4.9 μg/L with 5% significance and 90% power, allowing for 20% dropout, based on a standard deviation of 9.0 μg Se/L (standardised difference of 0.54). In a recent national survey, the mean plasma Se concentration among adult women in Malawi was 83.7 μg/L [4], and the trial is powered to increase Se concentration above the threshold for optimal activity of GPx3 (<84.9 μg/L).

Data on the number of participants (WRA and SAC) randomly assigned (with exclusions and reasons for exclusion), the flow of participants through enrolment, allocation to intervention, follow-up and analysis will be presented in a flow chart. The primary analysis will be carried out on groups as randomised (‘intention to treat’).

Tabulation of demographic data and other characteristics will be carried out by using the intention-to-treat datasets. No significance tests will be performed to test for differences at baseline. Descriptive statistics for continuous variables will include the mean, standard deviation, median, range and the number of observations. Categorical variables will be presented as numbers and percentages.

For the primary outcome, analysis of covariance (adjusting for baseline values) will be used to estimate a mean difference in Se status between the two arms of the trial. The results will be presented with a 95% confidence interval. For secondary outcomes, appropriate models will be used to examine the effect of the relevant intervention. Appropriate measures of effects will be reported with 95% confidence intervals.

Unadjusted and adjusted results will be presented for all analyses. Planned subgroup analyses for WRA will include 10-year age groups (20–29, 30–39 and 40–45 years), lactation status ('yes – exclusive breastfeeding'; 'yes – breastfeeding and complementary feeding'; 'not breastfeeding'), and pregnancy status (self-reported at end-line). All sub-group analyses will be performed by including a variable (or variables, as appropriate) for the sub-group and its interaction with the treatment in the model. Results will be interpreted with due caution. Full details of all analyses, including any additional covariates to be included in the adjusted models, will be set out *a priori* in a statistical analysis plan.

Although we have allowed for 20% missing data in the sample size calculations, only a small amount of missing data is expected and it is unlikely that it will have to be accounted for in any analysis. We would consider using multiple imputation if missing data were larger than expected or there were differential attrition between the trial arms. We would also attempt to ensure that the reason for the differential attrition was fully understood.

## Discussion

The AHHA Malawi trial is a pragmatic, community-based trial that will provide evidence on the efficacy of alleviating Se deficiency through consumption of maize flour enriched with Se through agro-biofortification. Agro-biofortification may be a cost-effective way to alleviate Se deficiency in Malawi; previous studies estimate a cost-per-alleviated case of about USD $0.36 per year [7]. The evidence from the trial may be used to inform future agriculture policy in Malawi and the wider region. The trial findings will be communicated to policy stakeholders and participating communities and reported in peer-reviewed journals.

### Trial status

Protocol version 4.2. Recruitment started on 26 June 2019 and was completed on 6 July 2019.

## Supplementary information

**Additional file 1.** Spirit checklist.

**Additional file 2.** a. Participant Information Sheet for adult women (English). b. Participant Information Sheet for adult women (Chichewa). c. Informed consent form for adult women (English). d. Informed consent form for adult women (Chichewa). e. Participant Information Sheet for the parent or guardian of schoolaged children (English). f. Participant Information Sheet for the parent or guardian of schoolaged children (Chichewa). g. Assent form for children (English). h. Assent form for children (Chichewa). i. Sample participant and maize flour recipient ID cards. A = Adult, C = Child, R = Recipient. Recipients are households in the study area but not participating in the trial. Recipient and Adult ID cards will be used at flour distribution points to ensure the correct allocation of flour for non-participant and participant households, respectively.

## Data Availability

Data sharing is not applicable to this article as no datasets were generated or analysed during the current study.

## Abbreviations

AE: Adverse event
AHHA: Addressing Hidden Hunger with Agronomy
DHS: Demographic and Health Survey
EA: Enumeration area
GPx3: Glutathione peroxidase 3
ICPMS: Inductively coupled plasma mass spectrometry
ID: Identifier
LUANAR: Lilongwe University of Agriculture and Natural Resources
ODK: Open Data Kit
PI: Principal investigator
RA: Research assistant
REC: Research ethics committee
SAC: School-age children
SAE: Serious adverse event
Se: Selenium
TA: Traditional authority
TSC: Trial steering committee
WRA: Women of reproductive age
Zn: Zinc
