# Supplementary material for: Can selenium deficiency in Malawi be alleviated through consumption of agro-biofortified maize flour? Study protocol for a randomised, double-blind, controlled trial
Source: Trials. 2019 Dec 30;20:795. doi: 10.1186/s13063-019-3894-2 (PMC6937860; doi:10.1186/s13063-019-3894-2)
Supplement: Supplementary file 2 — Additional file 2. a. Participant Information Sheet for adult women (English). b. Participant Information Sheet for adult women (Chichewa). c. Informed consent form for adult women (English). d. Informed consent form for adult women (Chichewa). e. Participant Information Sheet for the parent or guardian of schoolaged children (English). f. Participant Information Sheet for the parent or guardian of schoolaged children (Chichewa). g. Assent form for children (English). h. Assent form for children (Chichewa). i. Sample participant and maize flour recipient ID cards. A = Adult, C = Child, R = Recipient. Recipients are households in the study area but not participating in the trial. Recipient and Adult ID cards will be used at flour distribution points to ensure the correct allocation of flour for non-participant and participant households, respectively. [file 13063_2019_3894_MOESM2_ESM.zip › PublicationFiles-joy-et-al_appendix-2a_03-07-2019R1.docx]

# Additional file 2a. Participant Information Sheet for adult women (English).

Study name: Addressing Hidden Hunger with Agronomy (AHHA) Malawi.

Protocol version: 4.2

| Date | Participant ID [on copy kept by RA] |
| --- | --- |
|  |  |

*The following information should be provided to participant women (aged 20-45 years) prior to seeking consent. The information should be provided verbally with printed copies available on request.*

I am…………………………………………………………………… from …………………………………………………………………….

Introduction

We are conducting research in your village and neighbouring villages. Your participation is voluntary and you do not have to take part. Now we are going to provide you with information about the proposed research, so you can decide on your participation. Please ask questions if anything is not clear.

What is the purpose of the study?

The study aims to determine whether consumption of maize flour enriched with the micronutrient selenium delivers a health benefit. Selenium is a vital nutrient for the health of you and your children. Previous research has shown that selenium is typically deficient among populations in rural Malawi, including in Wimbe TA. We are looking at ways to sustainably increase consumption of selenium so that everyone can be healthier.

Why are we asking you to take part?

We have randomly selected villages in Wimbe TA to take part in this study. Every household within participating villages can be involved, if they wish. In your household there is at least one woman aged 20-45 years and at least one child aged 5-10 years. This means that your household is eligible for the trial. We have subsequently selected one eligible woman and one eligible child in your household to participate in the trial.

Do you have to take part?

You do not have to take part in the study.

What will happen if you take part?

Every household in the village will be provided free maize flour for 12 weeks, from early July through to early October this year. If your household takes part in the study then we will randomly assign you to one of two groups: flour enriched with selenium, or standard maize flour. We enriched the flour using fertilizers applied during the maize production. Maize was grown at Lilongwe University of Agriculture and Natural Resources, near Lilongwe. We will invite you and community members to visit the maize production site to see the process.

The flour will be distributed free of charge and we will request households to use this flour for their own consumption for the 12-week duration of the trial. Each household will be provided with enough flour to meet their daily requirements. Every 2 weeks we will arrange distribution of the maize flour to your household and we will monitor the consumption of the trial flour through observations and short questionnaires with the member of your household who is responsible for meal preparation.

To measure whether the flour has an effect on your nutritional status we will need to collect blood samples (6 mL) at the start and end of the trial. We will also collect blood samples from the selected school-aged child in your household, provided the child’s caregiver provides assent. These samples will be analysed in laboratories in Malawi and in the UK to measure the nutritional status of the participants so that we can determine whether the enriched flour has made a difference. The samples will only be analysed to provide information on the selenium status of the participant. Other parameters including HIV infection will not be tested.

What will you have to do?

We require the support of you and your community for this trial to work. We request that your households consumes the flour that they are provided with for the 12-week period, and we request that the trial flour is not sold or exchanged. For you, as a participating individual, we will conduct a short questionnaire including capturing information on your recent dietary consumption. We will then collect a blood sample (6 mL) at a mobile clinic in your village, at the start and end of the trial. Blood samples will be collected by trained nurses.

What are the possible benefits?

You and your household will receive free maize flour for a 12-week period. This will allow you to conserve your own stocks of maize for later in the year. Consumption of more selenium is likely to deliver health benefits and we might see some benefits occur during the short duration of the trial, such as lower incidence and duration of diarrhoea. However, we won’t know until the trial is complete whether you and your household are provided with the enriched flour or with control flour. Overall, the trial will deliver important information to researchers and the Malawi government. This information will inform decisions to improve nutrition through agriculture, including fertilizer recommendations.

What if something goes wrong?

We have conducted a thorough risk assessment and have plans in place to monitor and respond to any problems. There will be regular opportunities to meet the study team and you can let them know if you have any immediate concerns. If they are unable to address these concerns, then please contact Mr Leonard Banda, Lilongwe University of Agriculture and Natural Resources, P.O. Box 143, Lilongwe. 0997 69 29 87.

Can you change your mind about your household taking part?

You can change your mind and withdraw your consent to participate. Withdrawal can mean no further participation in the trial but continued receipt of control flour, or no further participation in the trial and no further receipt of flour, depending on your preference.

What will happen to the information generated during the trial?

The information we generate during the trial will be kept private. Only staff working on the trial will be able to access and use this information. Data will be anonymised, so any names and other identifying information will be removed before these datasets are shared.

Data will be hosted on a secure server accessible only to the study team. When data files are transferred they will be encrypted to ensure security. Data will be archived for a minimum of 10 years after the study end and will be destroyed once no longer needed.

What will happen to the results of this study?

This trial aims to determine the potential health benefits of consuming maize flour enriched with the selenium. We are planning to conduct this trial here and in neighbouring villages. The results of the trial will be used to inform policies and programmes in Malawi, including fertilizer recommendations. We will communicate the study findings to you and your community once the trial is complete.

Who has checked this study?

All research involving human participants is looked at by an independent group of people, called a Research Ethics Committee, to protect your interests. This study has been reviewed and approved by a committee at the College of Medicine, Blantyre, and by a committee at the London School of Hygiene & Tropical Medicine in the United Kingdom.

Further information and contact details

For further information about the trial or for any questions, please contact:

Mr Leonard Banda, Lilongwe University of Agriculture and Natural Resources, P.O. Box 143, Lilongwe. 0997 69 29 87

For further information about the ethical procedures related to this trial, or if you have any concerns about the conduct of the trial, please contact:

College of Medicine Research Ethics Committee, 3rd Floor, John Chiphangwi Learning Resource Centre, Private Bag 360, Chichiri, Blantyre. +265 (0)11 871 911
