# Supplementary material for: Can selenium deficiency in Malawi be alleviated through consumption of agro-biofortified maize flour? Study protocol for a randomised, double-blind, controlled trial
Source: Trials. 2019 Dec 30;20:795. doi: 10.1186/s13063-019-3894-2 (PMC6937860; doi:10.1186/s13063-019-3894-2)
Supplement: Supplementary file 2 — Additional file 2. a. Participant Information Sheet for adult women (English). b. Participant Information Sheet for adult women (Chichewa). c. Informed consent form for adult women (English). d. Informed consent form for adult women (Chichewa). e. Participant Information Sheet for the parent or guardian of schoolaged children (English). f. Participant Information Sheet for the parent or guardian of schoolaged children (Chichewa). g. Assent form for children (English). h. Assent form for children (Chichewa). i. Sample participant and maize flour recipient ID cards. A = Adult, C = Child, R = Recipient. Recipients are households in the study area but not participating in the trial. Recipient and Adult ID cards will be used at flour distribution points to ensure the correct allocation of flour for non-participant and participant households, respectively. [file 13063_2019_3894_MOESM2_ESM.zip › PublicationFiles-joy-et-al_appendix-2c_03-07-2019R1.docx]

# Additional file 2c. Informed consent form for adult women (English).

| **Statement** | **Initial or thumbprint** |
| --- | --- |
| I confirm that the information about the AHHA study (version 4.2) has been read to me and I understand it. I have had a chance to consider the information and any questions have been answered to my satisfaction. |  |
| I understand that my participation is voluntary and that individuals are free to withdraw at any time without giving any reason, without medical care or legal rights being affected. |  |
| I understand that data about me may be shared via a public data repository or by sharing directly with other researchers, and that all data will be anonymised so that people are not be identifiable from this information. |  |
| I agree to taking part in the AHHA study |  |

|  |  |  |
| --- | --- | --- |
| Printed name of participant | Signature of participant | Date |
|  |  |  |
| Printed name of witness* | Signature of witness | Date |

*If the participant is unable to read or write

I attest that I have explained the AHHA study information accurately, and to the best of my knowledge this was understood by the participant and that he/she has freely given their consent to participate* in the presence of the above named impartial witness (where applicable).

|  |  |  |
| --- | --- | --- |
| Printed name of researcher* | Signature of researcher | Date |

A copy of this consent form has been provided to the participant.
