# Supplementary material for: Can selenium deficiency in Malawi be alleviated through consumption of agro-biofortified maize flour? Study protocol for a randomised, double-blind, controlled trial
Source: Trials. 2019 Dec 30;20:795. doi: 10.1186/s13063-019-3894-2 (PMC6937860; doi:10.1186/s13063-019-3894-2)
Supplement: Supplementary file 2 — Additional file 2. a. Participant Information Sheet for adult women (English). b. Participant Information Sheet for adult women (Chichewa). c. Informed consent form for adult women (English). d. Informed consent form for adult women (Chichewa). e. Participant Information Sheet for the parent or guardian of schoolaged children (English). f. Participant Information Sheet for the parent or guardian of schoolaged children (Chichewa). g. Assent form for children (English). h. Assent form for children (Chichewa). i. Sample participant and maize flour recipient ID cards. A = Adult, C = Child, R = Recipient. Recipients are households in the study area but not participating in the trial. Recipient and Adult ID cards will be used at flour distribution points to ensure the correct allocation of flour for non-participant and participant households, respectively. [file 13063_2019_3894_MOESM2_ESM.zip › PublicationFiles-joy-et-al_appendix-2d_03-07-2019R1.docx]

# Additional file 2d. Informed consent form for adult women (Chichewa).

| **Mfundo** | **Sayini kapena chidindo** |
| --- | --- |
| Ndikuvomeleza kuti ndawerengeledwa unthenga okhudza kafukufuku ameneyu othetsa njala yobisika kudzera mu ulimi wachakudya (version 4.2) ndipo ndaumvetsetsa. Ndinali ndi mwayi wolingalira unthengawu ndipo mafunso ena aliwonse ayankhidwa ndipo ndakutitsidwa. |  |
| Ndamvetsetsa kuti kutenga nawo mbali kwanga ndipo ndili omasuka kusiya nthawi ina iliyonse osapelekanso chifukwa china chilichonse, komanso popanda kupheledwa ufulu olandira nthandizo lakuchipatala kapena ufulu wawo wovomelezeka wina ulionse. |  |
| Ndamvetsetsa kuti unthenga okhudza ine utha kugawidwa kudziko lonse kudzera njira zogawira unthenga kapena kuwapatsa opanga kafukufuku ena komanso unthenga onso udzapangidwa kukhala opanda dzina kapena chizindikiro change china chilichonse kuti anthu asadzathe kundizindikira kudzera muunthengawu |  |
| Ndavomera kuti nditenga nawo gawo mukafukufuku wothetsa njala yobisika kudzera mu ulimi wachakudya |  |

|  |  |  |
| --- | --- | --- |
| Dzina la wotenga nawo mbali | Sayini ya wotenga nawo mbali | Tsiku |
|  |  |  |
| Dzina la mboni* | Sayini ya mboni | Tsiku |

*Ngati wotenga nawo mbali samatha kuwerenga kapena kulemba

Ndikutsimikiza kuti ndafotokoza moyenera unthenga okhudza kafukufuku wothetsa njala yobisika kudzera mu ulimi wachakudya, ndipo mumkudziwa kwanga otenga nawo mbali amvetsetsa ndiponso apeleka chilolezo chawo kuti atenge nawo mbali.

|  |  |  |
| --- | --- | --- |
| Dzina lawopanga kafukufuku* | Sayini ya wopanga kafukufuku | Tsiku |

Pepala langati lomweli lapelekedwanso kwa wotenga nawo mbali.
