# Supplementary material for: Can selenium deficiency in Malawi be alleviated through consumption of agro-biofortified maize flour? Study protocol for a randomised, double-blind, controlled trial
Source: Trials. 2019 Dec 30;20:795. doi: 10.1186/s13063-019-3894-2 (PMC6937860; doi:10.1186/s13063-019-3894-2)
Supplement: Supplementary file 2 — Additional file 2. a. Participant Information Sheet for adult women (English). b. Participant Information Sheet for adult women (Chichewa). c. Informed consent form for adult women (English). d. Informed consent form for adult women (Chichewa). e. Participant Information Sheet for the parent or guardian of schoolaged children (English). f. Participant Information Sheet for the parent or guardian of schoolaged children (Chichewa). g. Assent form for children (English). h. Assent form for children (Chichewa). i. Sample participant and maize flour recipient ID cards. A = Adult, C = Child, R = Recipient. Recipients are households in the study area but not participating in the trial. Recipient and Adult ID cards will be used at flour distribution points to ensure the correct allocation of flour for non-participant and participant households, respectively. [file 13063_2019_3894_MOESM2_ESM.zip › PublicationFiles-joy-et-al_appendix-2f_03-07-2019R1.docx]

# Additional file 2f. Participant Information Sheet for the parent or guardian of school-aged children (Chichewa)

Study name: Addressing Hidden Hunger with Agronomy (AHHA) Malawi.

Protocol version: 4.2

| Date | Participant ID [on copy kept by RA] |
| --- | --- |
|  |  |

*Uthenga otsatirawu uyenera kupelekedwa kwa kholo kapena aliyense amene amayang’anira mwana amene akutenga nawo gawo (azaka 5-10) kumayambiliro kopempha chilolezo. Unthengawu upelekedwe pofototokoza ndipo mapepala a unthengawu atha kupatsidwa ngati apempha.*

Ine…………………………………………………………………… kuchokera ku…………………………………………………………….

Mawu woyambirira

Tikufuna kupanga kafukufuku mu m’mudzi wanu ndi midzi ina yoyandikana ndi mudzi wanu. Tikuyankhula ndi inu monga amene mumasamalira mwana amene atha kulowa mukafukufukuyu nthawi zambiri ndipo mwana amene mumamusamalirayu sakuyenera kutenga nawo gawo. Kutenga nawo gawo ndi kosakakamiza ndipo mwana amene mumamusamalirayu sakuyenera kutenga nawo gawo. Tsopano tikupatsani uthenga okhudza kafukufuku amene tikupempha kuti apangidweyu ndicholinga chakuti mupange chisankho chakuti mwana wanu atenga nawo mbali. Chonde funsani mafunso ngati penapake pasakumveka.

Kodi cholinga chakafukufukuyu ndi chiyani?

Kafukufukuyu akufuna kuwunika ngati kudya ufa ophatikiza ndi michere ina ngati selenium kumabweretsa phindu lokhudza umoyo. Selenium ndi michere yofunika ku umoyo wanu ndi wa ana anu. Kafukufuku wam’mbuyo waonetsa kuti michereyi ndiyopelewera mwa anthu ambiri akumudzi mu Malawi, ndi mwa TA Wimbe momwe. Tikuyang’ana njira zomwe tingapititsire patsogolo kadyedwe ka michereyi mopitilira ndicholinga chakuti aliyense akhale wa umoyo wabwino.

Nchifukwa chiyani tikupempha mwanayo kuti asamalire?

Tasankha mwachisawawa Midzi yomwe ili mwa TA Wimbe kuti itenge nawo gawo mukafukufukuyu. Khomo lina lililonse lomwe lili mu midzi yomwe kafukufukuyu akuchitika litha kutenga nawo mbali, ngati angafune. Pakhomo panu pali mwina mayi wazaka makumi awiri kulekeza zaka makumi anayi ndi zisanu ndi mwana m’modzi wa zaka zisanu kulekeza zaka khumi. Izi zikutanthauzi khomo lanu ndilololedwa kutenga nawo mbali mukafukufukyu. Tikuyankhula ndi inu monga woyang’anira pakhomo, ndipo tikupempha chilolezo chotenga nawo gawo mukafukufukuyu.

Kodi mwana amene mumamusamalirayu akuyenera kutenga nawo gawo?

Mwana wanu sakuyenera kutenga nawo mbali mukafukufukuyu.

Chidzachitike ndi chiyani ngati mwana wanu watenga nawo gawo?

Banja lina lililonse mu m’mudzi mwanu lidzalandira Ufa mwaulere kwa masabata khumi ndi awiri (12), kuyambira kumayambililo kwa mwezi wa July mpakana kumayambililo kwa mwezi wa October chaka chino. Ngat banja lanu latenga gawo mukafukufukuyu ndekuti lidzasankhidwa mwachisawawa ndikuikidwa ku gulu limodzi mwa magulu awiriwa: la ufa ophatikiza ndi Selenium kapena la Ufa osaphatikiza ndi chilichonse omwe umadyedwa nthawi zonse. Tidzaphatikiza michereyi pogwiritsa ntchito feteleza amene amathiridwa munthawi yaulimi wa chimanga. Chimanga chidzalimidwa ku Sukulu ya ukachenjede ya Ulimi ya LUANAR, yomwe ili kufupi ndi Lilongwe. Tidzakuitanani inuyo ndi anthu ena a m’mudzi mwanu kuti mudakayendere komwe kukulimidwa chimangacho ndikukaona ndondomeko yake.

Ufawu udzagawidwa mwaulere ndipo tidzapempha makomo kuti adzagwiritse ntchito ufawu pakudya kwa masabata khumi ndi awiri (12) akafukufukuyu. Khomo lililonse lidzapatsidwa ufa okwanira kadyedwe kawo ka tsiku ndi tsiku. Pa masabata awiri aliwonse tidzapanga kuti ufa upelekedwe ku banja lanu ndipo tidzaunikira ngati ufa wamu kafukufukuwu ukudyedwa kudzera mu kuwona ndi kugwiritsa ntchito mapepala amafunso ochepa kwa munthu m’modzi wapa banja lanu amene amakhudzidwa ndikuphika.

Kuti tiyeze ngati ufawu ukubweretsa kusintha ku nthanzi la mwana amene mumamusamalira tidzafunika kutenga ma sampulo a magazi (6 mL) kumayambiliro ndi kumathero kwa kafukufukuyu. Tidzatenganso sampulo ya magazi a mzimayi amene anakwanitsa dzaka dzakuti angabeleke pakhomo panupo. Masampulowa adzayezedwa ku malo oyezera kuti awunike mlingo wa nthanzi la wotenga nawo mbali ndicholinga chakuti tidziwe ngati ufa owonjexera micherewu wabweretsa kusintha. Ma sampulo amenewa adzawunikidwa kuti angopeleka unthenga okhudza mlingo Seleniyamu mwa otenga nawo mbali. Zinthu zina monga tizilombo toyambitsa matenda a EDZI sizidzayezedwa.

Kodi mwana wanu akuyenera kutani?

Tikupempha kudzipeleka kwanu ndi mwana wanu kutenga nawo gawo mukafukufuyu. Tikupempha kuti banja lanu lidzidya ufa omwe apatsidwe kwa masabata khumi ndi awiri (12), ndipo tikupempha kuti ufa umenewu usadzagulitsidwe kapena kusinthitsidwa. Kwa mwana wanu ngati munthu wotenga nawo gawo, tidzatenga sampulo ya magazi okwana (6 mL) ku chipatala choyendayenda m’mudzi mwanu. Tikukupemphani inuyo ngati osamalira mwanayu nthawi yambiri kuti muthandizire ndondomeko imeneyi pakumupelekeza mwanayu kuchipatala choyendayenda ndikuonetsetsa kuti akupatsidwa uthenga woyenera.

Kodi phindu lomwe lingapezeke ndi lanji?

Inuyo ndi Mudzi wanu mulandira ufa waulere kwanthawi yokwana masabata khumi ndi awiri (12). Izi zikulolani kusunga chimanga chanu ndikudzagwiritsa ntchito nthawi ina muchakachi. Kuwonjezera kupezeka kwa Seleniyamu kuli ndikuthekera kobweretsa phindu lokhudza umoyo wanu ndipo titha kuwona lina mwa phinduli likuchitika mu nthawi yochepa yakafukufukuyi, monga kutsika kwa matenda komanso nthawi yomwe matenda otsegula m’mimba amatenga kuti athe. Ngakhale zili choncho, sitingathe kudziwa pokhapokha kafukufukuyu atatha inu kapena banja lanu litamapatsidwa ufa owonjezeredwa ndi michere kapena ufa wa nthawi zones osaonjezera michere. Kuomba mkota, kafukufukuyu adzabweretsa unthenga ofunika kwa opanga kafukufuku ndi Boma la Malawi. Unthenga umenewu udzathandiza kupanga ziganizo zopititsira patsogolo thanzi kudzera mu ulimi, komanso kutsimikizira zafeteleza wabwino.

Nanga chinachake chitalakwika?

Tapanga kawuniwuni okwanira waziopsezo ndipo taika kale ndondomeko m’malo kuti zikathandizire kuyang’anira ndi kuthandizira mavuto ena aliwonse. Kudzikhala mwayi wapafupipafupi okumana ndi gulu la opanga kafukufukuyu ndipo mutha kuwadziwitsa ngati muli ndi zokhudza zina zili zones. Ngati sanathe kukuthandizani kumavuto anuwo, chonde lumikizanani ndi awa

Mr Leonard Banda, Lilongwe University of Agriculture and Natural Resources, P.O. Box 143, Lilongwe. 0997 69 29 87.

Mungasinthe maganizo okhudza kutenga nawo gawo kwanu?

Mutha kusintha maganizo anu ndi kulanda chilolezo chomwe munapeleka m’malo mwa banja lanu. Kulanda chilolezo kutha kutanthauza kusiya kutenga nawo gawo mukafukufuku koma kupitiliza kulandira ufa osaphatikiza ndi michere, kapena kusiya kutenga nawo gawo mukafukufuku ndi kusiya kulandira ufa, kutengera ndi m’mene inu mwasankhira.

Kodi chidzachitike ndi chiyani ku unthenga omwe upezedwe mukafukufuku ameneyu?

Unthenga omwe upezedwe mukafukufuku ameneyu udzasungidwa mwachinsinsi. Amene adzakhale ndi mwayi owona kapena kugwiritsa ntchito unthengawu ndi okhawo ogwira ntchito nafe. Unthengawu udzakhala wachinsinsi, kotero dzina lanu ndi unthenga wanu wina omwe munthu angakudziwireni udzachotsedwapo unthengawu usanagawidwe

Unthenga udzasungidwa pamakina otetezedwa amene angagwiridwe ndi okhawo okhudzidwa ndi kafukufukuyu. Pamene ma fayilo a unthenga azatumizidwe kumakinawa adzabisidwa mumakinamo kuti tidzaonetsetse kuti unthengawu ukutetezedwa. Unthengawu udzasungidwa kwa dzaka dzosachepera khumikuchokera pamene adzathere kafukufukuyu ndipo udzatayidwa mosamala ngati sudzafunikanso.

Chidzachitike ndi chiyani kuzotsatira zakafukufukuyu?

Kafukufukuyu akufuna kufufuza phindu lokhudza umoyo lomwe lingapezeke pakudya ufa ophatikiza ndi michere ya seleniyamu. Tikulingalira kupanga kafukufukuyu kuno ndi midzi ina yoyandikana nanu. Zotsatira zakafukufukuyu zitsagwiritsidwa ntchito popeleka umboni ku malamulo ndi mapologalamu aku Malawi, komanso ndemanga zokhudza fetelezawofunika. Tidzakudziwitsani inuyo ndi mudzi wanu za zotsatira zakafukufukuyu pamene kafukufuku wamalizidwa.

Awunika kafukufuyu ndi ndani?

Kafukufuku wina aliyense okhudza anthu amawunikidwa ndi gulu loyima palokha lotchedwa Research Ethics Committee lomwe limateteza udulu wanu. Kafukufukuyu wawunikidwa ndi kulolezedwa ndi komiti yomwe ili ku sukulu yaukachenjede ya College of Medicine ku Blantyre, komanso sukulu ya Ukachenjede ya London School of Hygiene & Tropical Medicine yomwe ili kunja kwa dziko lino ku United Kingdom.

Unthenga owonjezera ndi wothandizira kulumizana

Mukafuna kudziwa zambiri zakafukufukuyu kapena mukakhala ndi mafunso, chonde lumikizanani ndi:

Mr Leonard Banda, Lilongwe University of Agriculture and Natural Resources, P.O. Box 143, Lilongwe. 0997 69 29 87.

Mukafuna kudziwa zambiri zokhudzana ndondomeko yokhudzana ndi kayendetsedwe ka kafukufukuyu, kapena mukakhala ndi madandaulo aliwonse okhudza kafukufukuyu chonde lumikizananani ndi:

College of Medicine Research Ethics Committee, 3rd Floor, John Chiphangwi Learning Resource Centre, Private Bag 360, Chichiri, Blantyre. +265 (0)11 871 911
