# Supplementary material for: Can selenium deficiency in Malawi be alleviated through consumption of agro-biofortified maize flour? Study protocol for a randomised, double-blind, controlled trial
Source: Trials. 2019 Dec 30;20:795. doi: 10.1186/s13063-019-3894-2 (PMC6937860; doi:10.1186/s13063-019-3894-2)
Supplement: Supplementary file 2 — Additional file 2. a. Participant Information Sheet for adult women (English). b. Participant Information Sheet for adult women (Chichewa). c. Informed consent form for adult women (English). d. Informed consent form for adult women (Chichewa). e. Participant Information Sheet for the parent or guardian of schoolaged children (English). f. Participant Information Sheet for the parent or guardian of schoolaged children (Chichewa). g. Assent form for children (English). h. Assent form for children (Chichewa). i. Sample participant and maize flour recipient ID cards. A = Adult, C = Child, R = Recipient. Recipients are households in the study area but not participating in the trial. Recipient and Adult ID cards will be used at flour distribution points to ensure the correct allocation of flour for non-participant and participant households, respectively. [file 13063_2019_3894_MOESM2_ESM.zip › PublicationFiles-joy-et-al_appendix-2g_03-07-2019R1.docx]

# Additional file 2g. Assent form for children (English)

| **Child/young person (or if unable to, parent on their behalf) to complete** | **Please circle all you agree with:** | |
| --- | --- | --- |
| Has information about this project been read to you? | Yes | No |
| Do you understand what this project is about? | Yes | No |
| Have you had any questions answered in a way you understand? | Yes | No |
| Do you understand that it is ok to stop taking part at any time? | Yes | No |
| Are you happy to take part? | Yes | No |

If you would like to take part in this project please write your name and today’s date:

|  |  |
| --- | --- |
| Your name | Date |

Your parent or guardian must write their name here too if they are happy for you to take part:

|  |  |  |
| --- | --- | --- |
| Name of parent/guardian | Signature of parent/guardian | Date |

The researcher who explained this project to you needs to sign too:

|  |  |  |
| --- | --- | --- |
| Printed name of researcher* | Signature of researcher | Date |

A copy of this consent form has been provided to the participant.
