# Supplementary material for: Can selenium deficiency in Malawi be alleviated through consumption of agro-biofortified maize flour? Study protocol for a randomised, double-blind, controlled trial
Source: Trials. 2019 Dec 30;20:795. doi: 10.1186/s13063-019-3894-2 (PMC6937860; doi:10.1186/s13063-019-3894-2)
Supplement: Supplementary file 2 — Additional file 2. a. Participant Information Sheet for adult women (English). b. Participant Information Sheet for adult women (Chichewa). c. Informed consent form for adult women (English). d. Informed consent form for adult women (Chichewa). e. Participant Information Sheet for the parent or guardian of schoolaged children (English). f. Participant Information Sheet for the parent or guardian of schoolaged children (Chichewa). g. Assent form for children (English). h. Assent form for children (Chichewa). i. Sample participant and maize flour recipient ID cards. A = Adult, C = Child, R = Recipient. Recipients are households in the study area but not participating in the trial. Recipient and Adult ID cards will be used at flour distribution points to ensure the correct allocation of flour for non-participant and participant households, respectively. [file 13063_2019_3894_MOESM2_ESM.zip › PublicationFiles-joy-et-al_appendix-2h_03-07-2019R1.docx]

# Additional file 2h. Assent form for children (Chichewa)

| **Mwana/ wachinyamata (ngati sangathe, uzani kholo m’malo mwake) kuti lipitilize** | **Chonde zungulizani zomwe mukugwirizana nazo:** | |
| --- | --- | --- |
| Kodi akuwelengerani unthenga okhudza kafukufukuyu? | Eya | Ayi |
| Kodi mwamvetsetsa kuti kafukufukuyi ndi yokhudza chiyani? | Eya | Ayi |
| Kodi mwayankhidwa funso lina lililonse munjira yakuti mwamvetsetsa? | Eya | Ayi |
| Mukumvetsetsa kuti ndizabwinobwino kusiya kutenga nawo mbali nthawi ina iliyonse? | Eya | Ayi |
| Muli osangalala kutenga nawo mbali? | Eya | Ayi |

Ngati mukufuna kutenga gawo mu kafukufukuyi chonde lembani dzina lanu ndi tsiku la lero:

|  |  |
| --- | --- |
| Dzina lanu | Tsiku |

Makolo anu kapena okuyang’anirani ayenera kulemba dzina lawo ngati ali osangalatsidwa kutenga gawo:

|  |  |  |
| --- | --- | --- |
| Dzina la kholo/ woyan’ganira mwana | Sayini ya kholo/woyan’ganira mwana | Tsiku |

Wopanga kafukufuku amene wakufotokerani za pulojekitiyi aynene kusayinanso:

|  |  |  |
| --- | --- | --- |
| *Dzina laopanga kafukufuku* | Sayini ya opaanga kafukufuku | Tsiku |

Pepala langati lomweli lapelekedwanso kwa wotenga nawo mbali.
