# Supplementary material for: Can selenium deficiency in Malawi be alleviated through consumption of agro-biofortified maize flour? Study protocol for a randomised, double-blind, controlled trial
Source: Trials. 2019 Dec 30;20:795. doi: 10.1186/s13063-019-3894-2 (PMC6937860; doi:10.1186/s13063-019-3894-2)

# Additional file 2i. Sample participant and maize flour recipient ID cards. A = Adult, C = Child, R = Recipient. Recipients are households in the study area but not participating in the trial. Recipient and Adult ID cards will be used at flour distribution points to ensure the correct allocation of flour for non-participant and participant households, respectively.


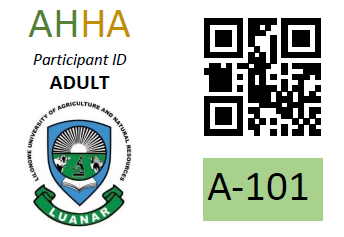


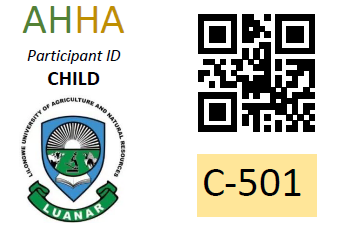


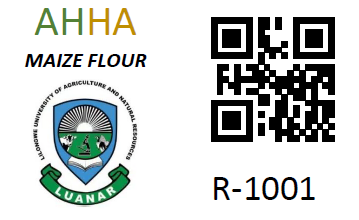


Reverse of all cards:


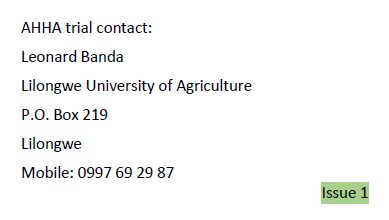

Supplement: Supplementary file 2 — Additional file 2. a. Participant Information Sheet for adult women (English). b. Participant Information Sheet for adult women (Chichewa). c. Informed consent form for adult women (English). d. Informed consent form for adult women (Chichewa). e. Participant Information Sheet for the parent or guardian of schoolaged children (English). f. Participant Information Sheet for the parent or guardian of schoolaged children (Chichewa). g. Assent form for children (English). h. Assent form for children (Chichewa). i. Sample participant and maize flour recipient ID cards. A = Adult, C = Child, R = Recipient. Recipients are households in the study area but not participating in the trial. Recipient and Adult ID cards will be used at flour distribution points to ensure the correct allocation of flour for non-participant and participant households, respectively. [file 13063_2019_3894_MOESM2_ESM.zip › PublicationFiles-joy-et-al_appendix-2i_03-07-2019R1.docx]
